# *KIAA2022/NEXMIF* c.1882C>T (p.Arg628*) Variant in a Romanian Patient with Neurodevelopmental Disorders and Epilepsy: A Case Report and Systematic Review

**DOI:** 10.3390/life15030497

**Published:** 2025-03-19

**Authors:** Catalina Mihaela Anastasescu, Veronica Gheorman, Simona Viorica Godeanu, Adriana Cojocaru, Floris Petru Iliuta, Mioara Desdemona Stepan, Victor Gheorman

**Affiliations:** 1 Children’s Mental Health Center, Hospital of Neuropsychiatry Craiova, 200349 Craiova, Romania; catalina_tocea@yahoo.com (C.M.A.); godeanusimona@yahoo.com (S.V.G.); 2 Department of Medical Semiology, University of Medicine and Pharmacy of Craiova, 200349 Craiova, Romania; 3 Department of Neurosciences, “Victor Babes” University of Medicine and Pharmacy, 2 Eftimie Murgu Square, 300041 Timisoara, Romania; adriana.cojocaru@umft.ro; 4 Department of Psychiatry and Psychology, Discipline of Psychiatry, Faculty of Dental Medicine, “Carol Davila” University of Medicine and Pharmacy, 010221 Bucharest, Romania; floris.iliuta@umfcd.ro; 5 Department of Psychiatry, “Prof. Dr. Alexandru Obregia” Clinical Hospital of Psychiatry, 041914 Bucharest, Romania; 6 Department of Infant Care-Pediatrics-Neonatology, University of Medicine and Pharmacy of Craiova, 200349 Craiova, Romania; desdemona.stepan@umfcv.ro; 7 Department of Psychiatry, University of Medicine and Pharmacy of Craiova, 200349 Craiova, Romania; victor.gheorman@umfcv.ro

**Keywords:** *NEXMIF* gene pathogenic variant, neurodevelopmental disorders, epilepsy

## Abstract

Pathogenic variants in the *NEXMIF* gene are associated with a broad neurodevelopmental phenotype, including autism spectrum disorder (ASD), intellectual disability (ID), and epilepsy. However, the role of *NEXMIF* in specific epileptic syndromes remains insufficiently explored. We present the case of an 11.9-year-old Romanian girl diagnosed with ASD, attention-deficit/hyperactivity disorder (ADHD), mild ID, and Jeavons syndrome (generalized epilepsy characterized by eyelid myoclonia, absence seizures, and photosensitivity). Genetic testing identified a pathogenic *NEXMIF* variant: c.1882C>T (p.Arg628*), a pathogenic variant rarely reported in the literature, with only two documented cases to date. To better understand the genotype–phenotype correlation, we conducted a systematic review of *NEXMIF*-associated disorders and compared our findings with previously reported cases. Our analysis suggests that *NEXMIF* variants may contribute to a broader spectrum of epileptic syndromes, including photosensitive epilepsy such as Jeavons syndrome. This highlights the need for a greater awareness of atypical seizure presentations in individuals with *NEXMIF*-related disorders. This study underscores the importance of genetic testing in individuals with overlapping ASD and epilepsy phenotypes as early diagnosis may facilitate targeted therapeutic interventions and genetic counseling. Further research is needed to clarify the molecular mechanisms linking *NEXMIF* dysfunction to epileptic syndromes and neurodevelopmental disorders.

## 1. Introduction

### 1.1. The Role of NEXMIF in Neurodevelopmental Disorders

Pathogenic variants in the *NEXMIF* gene, formerly known as *KIAA2022*, *XLID98*, or *XPN*, have been identified as a cause of X-linked intellectual disability 98 (*XLID98*), a neurodevelopmental disorder with a complex phenotypic spectrum [1,2,3]. *NEXMIF*, located on chromosome Xq13.2, encodes a protein with a still poorly understood biological function, although emerging evidence suggests a pivotal role in neuronal development and synaptic plasticity.

Pathogenic *NEXMIF* variants are associated with autism spectrum disorder (ASD), epilepsy, strabismus, postnatal growth retardation, and microcephaly. Phenotypic expression varies significantly, with males frequently exhibiting autistic traits, while female carriers may present with variable severity or remain asymptomatic, suggesting an influence of X-inactivation on phenotypic expressivity [4,5]. The clinical manifestations appear to correlate with the degree of *NEXMIF* loss, reinforcing its critical role in neuronal function [4].

At the molecular level, *NEXMIF* loss-of-function variants impair neuronal development. Studies on rat hippocampal neurons revealed that *NEXMIF* knockdown leads to significant deficits in neurite outgrowth, affecting both dendrites and axons, suggesting a crucial role in neuronal morphogenesis [6,7,8]. Further, in vitro studies indicate that *NEXMIF* is highly expressed in fetal and adult murine brains, supporting its role in postmitotic neuronal maturation [9]. These findings suggest that *NEXMIF* dysfunction contributes to neurodevelopmental disorders, yet its precise involvement in ASD pathophysiology remains incompletely understood [10]. Transient expression studies further support its involvement in neurite extension and neuronal circuit formation, implicating *NEXMIF* in the pathogenesis of intellectual disability [11,12].

### 1.2. Autism Spectrum Disorder and Jeavons Syndrome: A Complex Comorbidity

ASD is a heterogeneous neurodevelopmental disorder characterized by deficits in social communication, restricted interests, and repetitive behaviors that emerge early in life and persist throughout development [13]. It is hypothesized that the disruption of brain development and also synaptic alterations lead to ASD-specific behaviors [7,14,15].

The specific causes of ASD remain unknown. However, ASD is a complex disorder characterized by a wide spectrum of symptoms and severity, with both genetic and non-genetic factors playing a significant role. Additionally, neuroinflammatory and metabolic mechanisms have been the focus of extensive research. While growing evidence suggests that inflammation may contribute to psychiatric disorders, including ASD, the precise pathogenic mechanisms of neuroinflammation in ASD continue to be actively investigated and remain incompletely understood [16,17,18,19]. Proinflammatory cytokines regulate gene network [17]. Genetic anomalies are associated with syndromic ASD [20,21], while non-syndromic ASD may be determined by prenatal and postnatal environmental factors (parental health including mental disorders, infections, medications, and drug and alcohol abuse) and remains idiopathic [14,22,23,24,25,26,27,28,29].

Jeavons syndrome, or eyelid myoclonia with absences (EMA), is an idiopathic generalized epilepsy syndrome typically presenting between 6 and 8 years of age, predominantly affecting females. It is characterized by eyelid myoclonia, photosensitivity, and absence seizures, often triggered by eye closure [30,31,32,33]. Seizures in Jeavons syndrome can be highly refractory, occurring dozens to hundreds of times per day. While the occipital cortex has been implicated in its pathophysiology, recent studies suggest epileptogenic activity extending to the posterior temporal cortex [34,35,36].

Genetic studies have identified several candidate genes associated with Jeavons syndrome, including *SYNGAP1*, *NEXMIF*, *RORB*, *NAA10*, and *CHD2* [31,34,35]. X-linked *NEXMIF* variants have been strongly associated with a broad spectrum of epilepsy [37]. Furthermore, a study conducted by Ye Z.L. et al. in 2024 highlights that different NEXMIF variants are linked to intellectual disability, with some of these variants also being associated with epilepsy, either in the presence or absence of intellectual disability [38]

### 1.3. Case Report Rationale

We present the case of an 11.9-year-old girl diagnosed with ASD, Attention Deficit Hyperactivity Disorder (ADHD), Mixed Learning Disorder, Mild Intellectual Disability, and absence with eyelid myoclonic seizures (known as Jeavons syndrome). Given the complexity of her clinical presentation, genetic testing was pursued, leading to the identification of a *NEXMIF* variant. This case contributes to the growing body of evidence linking *NEXMIF* variants to neurodevelopmental disorders and epilepsy, aiming to contextualize our findings within the existing literature and highlight the phenotypic variability associated with these variants.

Furthermore, our findings emphasize the necessity of genetic testing in patients with ASD and epilepsy, particularly those with drug-resistant or unusual seizure types, to improve early diagnosis and individualized management.

## 2. Materials and Methods

### 2.1. Informed Consent

The study protocol was approved by University and Scientific Ethics and Deontology Commission of the University of Medicine and Pharmacy in Craiova, Romania according to decision No. 154/24 September 2021 and adhered to the principles outlined in the Declaration of Helsinki and the Code of University Ethics. We obtained the informed consent from the patient’s parents for publishing the data.

### 2.2. Genetic Analysis

To investigate the genetic basis of the patient’s neurodevelopmental phenotype, a two-step molecular genetic analysis was conducted. The test was performed by the Dolj Regional Center for Medical Genetics from Craiova, Romania.

Initially, molecular genetic testing for autism was conducted using MLPA (Multiplex Ligation-dependent Probe Amplification by MRC-Holland, Amsterdam, The Netherlands) with the genetic analyzer ABI Prism 3730 (Applied Biosystems). The SALSA MLPA probemix P343 Autism 1 kit (MRC-Holland, Amsterdam, The Netherlands), targeting the UBE3A, GABRB3, and SHANK3 genes was used. This analysis identifies deletions or duplications in the 15q11–q13 region, including alterations in the UBE3A and GABRB3 genes, as well as a microdeletion in the 15q13 region encompassing CHRNA7 and microdeletion in the 16q11 and 22q13, affecting the SHANK3 gene.

Subsequently, next-generation sequencing (NGS)-based genetic testing was performed using the Invitae sequencing platform. This comprehensive approach included sequence analysis and deletion/duplication testing of 930 genes. This panel incorporated the Invitae Epilepsy Panel, Invitae Leukodystrophy and Genetic Leukoencephalopathy Panel, and Invitae Neurodevelopmental Disorders (NDD) Panel. Genomic DNA was extracted from the collected samples and subjected to targeted region enrichment using a hybridization-based protocol. Sequencing was performed using Illumina technology, ensuring high-depth coverage (≥50× for all targeted regions), with additional analysis applied when necessary. The validation study confirmed a >99% analytical sensitivity and specificity for the detection of single nucleotide variants (SNVs), insertions, and deletions (<15 bp), as well as exon-level deletions and duplications.

This methodological approach ensured a highly sensitive and specific assessment of potential pathogenic variants contributing to the patient’s clinical presentation.

### 2.3. Systematic Literature Review

This study adhered to the Preferred Reporting Items for Systematic Reviews and Meta-Analyses (PRISMA) guidelines [39].

Our primary objective was to contextualize a rare case of *NEXMIF* variant associated with Jeavons syndrome and autistic features by comparing it with previously reported cases and synthesizing the best available data.

To identify the relevant literature, a comprehensive search was conducted in PubMed and Google Scholar. The search strategy targeted studies involving the *NEXMIF* gene and its alternative names, using the following key terms:

- Gene-Specific Searches: “*NEXMIF* cases”, “*KIAA2022* cases”, “*KIDLIA* cases”, and “*XPN* cases”;
- Pathogenic Variant-Specific Searches: “*NEXMIF* c.1882C>T p.Arg628*”, “*KIAA2022* c.1882C>T p.Arg628*”, “*KIDLIA* c.1882C>T p.Arg628*”, and “*XPN* c.1882C>T p.Arg628*”;
- Phenotype-Specific Searches: “*NEXMIF* autism”, “*NEXMIF* autism Jeavons”, “*KIAA2022* autism”, “*KIAA2022* autism Jeavons”, “*KIDLIA* autism”, “*KIDLIA* autism Jeavons”, “X*PN* autism”, and “*XPN* autism Jeavons”

Study Selection Process. The article screening process was conducted systematically by two independent reviewers, following predefined inclusion and exclusion criteria. We included studies reporting the same *NEXMIF* pathogenic variant as in our case, longitudinal studies, cohort studies, case reports, and peer-reviewed articles published in English. We excluded studies unrelated to the *NEXMIF* c.1882C>T (p.Arg628*) variant, duplicate publications, articles lacking clear clinical outcomes or insufficient methodological details, non-peer-reviewed literature (preprints, books, and congress presentations), articles published in languages other than English. Non-peer-reviewed studies were excluded to ensure methodological rigor and reliability of reported cases.

This systematic approach allowed for the identification of high-quality evidence, ensuring an accurate comparison with previously documented *NEXMIF* variants.

**Figure 1:**
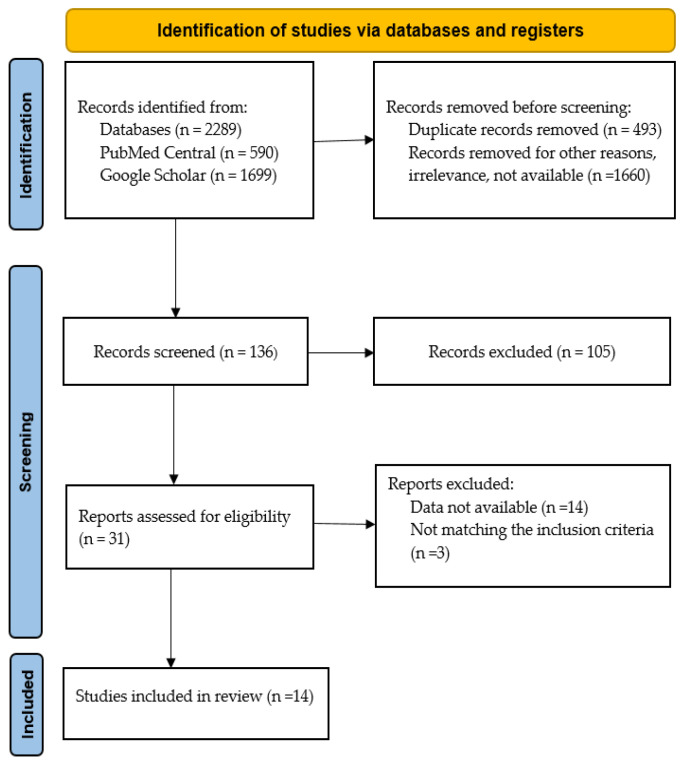
The flow chart of the systematic review on *NEXMIF* c.1882C>T (p.Arg628*).

To ensure transparency, the selection workflow was mapped using a PRISMA flow diagram (Figure 1).

## 3. Results

### 3.1. Case Report

#### 3.1.1. Clinical Presentation

The subject of this case report is an 11.9-year-old Romanian girl, the first and only child of her mother. The pregnancy was complicated by threatened abortion, which was managed with pharmacological treatment, including Butylscopolammonium bromide (Scobutil) and Drotaverine hydrochloride (No-SPA), as recalled by the mother. Additionally, the mother was diagnosed with uterine fibroids during pregnancy.

A retrospective assessment of maternal emotional status, based on detailed anamnesis and implementation of the Hamilton Scale, suggested the presence of anxiety and depressive symptoms during pregnancy. However, there was no other reported family history of psychiatric, neurological, or genetic disorders.

The patient was delivered via cesarean section at 39 weeks of gestation, with a birth weight of 3350 g. No perinatal complications were reported. Early motor development was within the expected parameters: she achieved head control at 2–3 months, sat without support at 6–7 months, and walked independently by 12 months. The mother reported that she began vocalizing syllables at 7–8 months.

Despite these early developmental milestones, a significant delay in language acquisition was observed. By 2.5 years of age, the patient could produce only a few meaningful words, did not form sentences, but demonstrated an ability to understand simple verbal commands. Cognitive development was below the expected level for each age stage.

Difficulties in social integration were evident from early childhood, characterized by challenges in communication, social adaptation, behavioral rigidity, sensory desynchronization, and atypical interactions. These features raised concerns regarding a neurodevelopmental disorder, prompting further clinical evaluation and genetic testing.

At the age of 6 years, the patient was referred for psychiatric evaluation due to persistent difficulties in social adaptation and integration, along with language and behavioral abnormalities.

The psychopathological profile included the following:

- Atypical language development: peculiar prosody (high-pitched tone), stereotyped verbal expressions, poor articulation, and limited sentence structure.
- Restricted and repetitive behaviors: solitary play patterns and fixations on specific objects (e.g., an intense attraction to earrings and hair, with a compulsion to touch them).
- Social and behavioral challenges: inappropriate social interactions, difficulty understanding social norms, and the presence of imaginary friends.
- Cognitive and executive dysfunction: deficits in attention, working memory, and executive functions, alongside fine motor impairments.

Following a comprehensive psychiatric assessment, the patient was diagnosed with: autism spectrum disorder (ASD), Attention Deficit/Hyperactivity Disorder (ADHD), Mild Intellectual Disability. These findings highlighted a complex neurodevelopmental profile, warranting further investigation, including genetic testing, to elucidate potential underlying etiologies.

**Figure 2:**
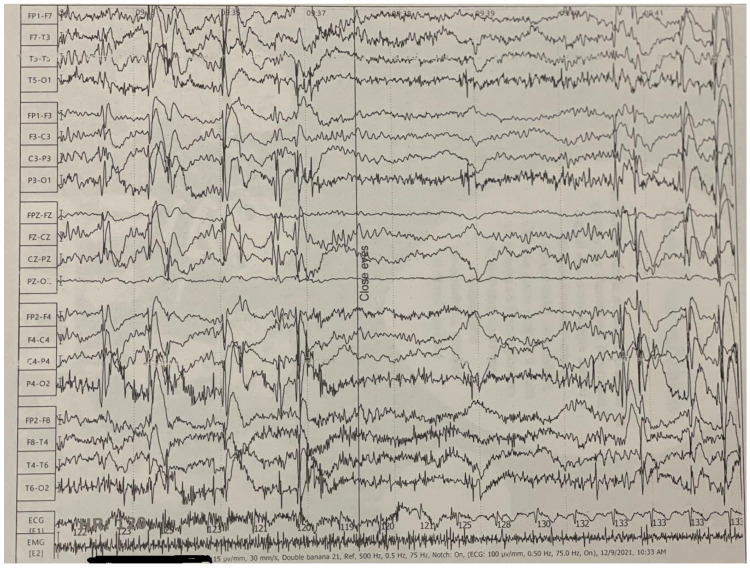
Electroencephalographic (EEG) findings in our patient. EEG recording in the awake state reveals generalized polyspike wave (3–6 HZ) elicited by eye closure and intermittent photic stimulation and eyelid myoclonia. Although the seizures were therapeutically controlled, the EEG shows the same abnormal pattern.

At the age of 8, the patient was additionally diagnosed with a disturbance in the development of scholastic skills, with a particular deficit in arithmetic calculation, reflecting difficulties in acquiring age-appropriate academic knowledge. Comprehensive ophthalmological and cardiological evaluations did not reveal any abnormalities. At the age of 9, following a detailed neurological assessment, the patient was diagnosed with Jeavons syndrome, characterized by palpebral myoclonus and epileptic absences and photosensitivity, which were clinically evident. Electroencephalographic (EEG) findings were consistent with this diagnosis, demonstrating characteristic features of Jeavons syndrome (Figure 2). The first choice of treatment for the patient was Levetiracetam, but we did not obtain the control of the seizures. Later, Levetiracetam was associated with Sodium Valproat. After this association, the seizures were controlled, but the EEG continued to show polyspike waves.

#### 3.1.2. Biologic Assessment

Comprehensive laboratory investigations revealed that blood count, as well as serum levels of copper, zinc, selenium, iron, magnesium, interleukin-6 (IL-6), interleukin-8 (IL-8), and tumor necrosis factor-alpha (TNF-α), were within normal limits. Amino acid profiling was conducted in both serum and urine.

Urinary amino acid analysis demonstrated deviations from reference values, including decreased levels of methylhistidine (76 µmol/g; reference range: 96–1959 µmol/g), lysine (16 µmol/g; reference range: 31–394 µmol/g), cystine (21 µmol/g; reference range: 26–98 µmol/g), asparagine (36 µmol/g; reference range: 49–466 µmol/g), and aminoadipic acid (14 µmol/g; reference range: 15–271 µmol/g).

In serum, a slight increase in glutamine levels was observed (709 µmol/L; reference range: 410–700 µmol/L).

#### 3.1.3. Phenotypic Features

**Figure 3:**
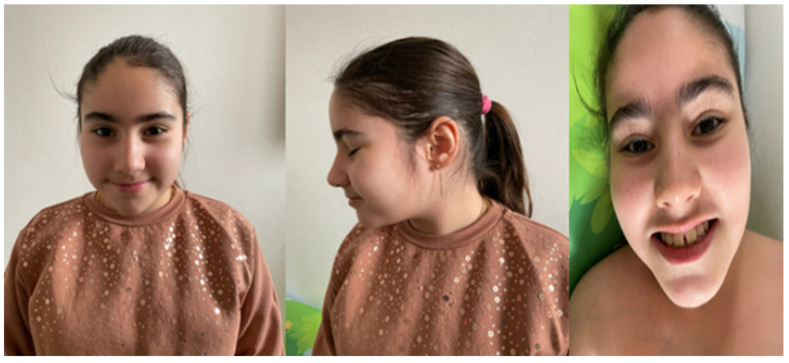
Facial features of a 11.9-year-old Romanian girl. The patient presents with a frequent smiling expression and dysmorphic features that include a narrow forehead, hooded eyelids, subtle ear abnormalities (lower set ear, prominent antihelix stem, inferior antihelix cleft, and prominent antitragus), a hypotonic mouth, and a thin upper lip.

The patient exhibits a characteristic facial phenotype and dysmorphic features. These phenotypic traits are consistent with previously reported descriptions in the literature [1] (Figure 3).

#### 3.1.4. Genetic Analysis Results

The result of MLPA test was negative.

At the INVITAE diagnostic testing results identified a pathogenic variant in the *NEXMIF* gene (c.1882C>T; p.Arg628*). We cannot state with certainty if this variant is de novo for our patient because the family of the patient was not tested. Additionally, several variants of uncertain significance (VUS) were detected including c.3460G>A (p.Val1154Met) in *CACNA1B*, c.1883A>C (p.Glu628Ala) in CSF1R, c.1504G>A (p.Ala502Thr) in *KCNQ2*, c.730+5G>A (intronic) in *MAN1B1*, c.1010C>T (p.Pro337Leu) in *MED17*, and a gain of the entire coding sequence of *PRODH.* NEXMIF variant and all the VUS were in a heterozygous form. Furthermore, benign variants associated with pseudodeficiency alleles were identified, including c.2065G>A (p.Glu689Lys) in *GAA* (Exon 15) and c.1685T>C (p.Ile562Thr) in *GALC* (Exon 15), both in heterozygous form.

According to ClinVar, a database hosted by the National Center for Biotechnology Information (NCBI), the p.Arg628* variant in the *NEXMIF* gene introduces a premature translational stop codon. This alteration is absent from population databases, including GnomAD (no reported frequency), and has been classified as pathogenic. The variant is reported to result in an absent or disrupted protein product. Loss-of-function variants in *NEXMIF* are well established as pathogenic [40].

### 3.2. Review of the Literature on NEXMIF c.1882C>T (p.Arg628*)

Following the genetic analysis of our patient, we considered that the identified heterozygous pathogenic variant in *NEXMIF* (c.1882C>T; p.Arg628*) likely contributes to the patient’s clinical phenotype. The patient exhibits features consistent with moderate impairment, significantly affecting her quality of life. She experiences social integration difficulties, with pronounced autistic traits and comorbid Attention Deficit/Hyperactivity Disorder (ADHD). Additionally, she struggles to meet academic requirements, presenting with Mild Intellectual Disability. Her epileptic seizures are only partially controlled with medication.

To contextualize our findings, we conducted a literature review to identify previously reported cases of this pathogenic variant and to compare our case with others harboring different *NEXMIF* variants.

This variant is cataloged as pathogenic in ClinVar under Variation ID: 190220 with the following identifiers [40,41]:

- Transcript: NM_001008537.3 (*NEXMIF*)*:* c.1882C>T (p.Arg628Ter);
- Nucleotide Change: NM_001008537.3:c.1882C>T;
- Protein Change: NP_001008537.1:p.Arg628Ter (R628*);
- Variant Classification: single-nucleotide variant (1 bp length);
- Genomic Location: Xq13.3 (X: 74,742,675 [GRCh38]; X: 73,962,510 [GRCh37]).

A search for the p.Arg628* variant of *NEXMIF* in the GnomAD v4.1.0 database identified three variant IDs: X-74742674-C-CA (exome), X-74742674-C-T (present in both exome and genome datasets), and X-74742675-G-T (exome) (Table 1).

A systematic literature search was conducted using PubMed Central and Google Scholar. Articles that were not relevant to our research focus or represented duplicates were excluded. For this review, we included two articles specifically describing the *NEXMIF* c.1882C>T (p.Arg628*) variant. Our analysis identified two patients previously reported with the c.1882C>T (p.Arg628*) variant (Table 2).

Following our review of cases in the literature reporting the *NEXMIF* c.1882C>T (p.Arg628*) variant, we analyzed 12 additional studies describing different *NEXMIF* variants to compare our findings with previously reported variants. These studies, published between 2017 and 2024, provided valuable insights into the genotype–phenotype correlations of *NEXMIF*-related disorders. The comparative analysis of genetic findings and associated clinical manifestations is summarized in Table 3.

## 4. Discussion

### 4.1. Genotype–Phenotype Correlations in NEXMIF Variants

Pathogenic variants in the *NEXMIF* gene have been increasingly recognized as a cause of neurodevelopmental disorders, with variable expressivity depending on the specific variant, the sex of the individual, and potential modifying genetic or environmental factors.

The *NEXMIF* gene was initially identified by Cantagrel et al. (2004) at the Xq13.2 breakpoint in a pericentric inversion of the X chromosome. The study reported two males with severe X-linked intellectual disability (XLID), while a carrier female remained asymptomatic, suggesting that the loss of *NEXMIF* function leads to a severe phenotype in hemizygous males [12]. Subsequent studies confirmed the role of *NEXMIF* variants in XLID and autism spectrum disorder (ASD), with reported phenotypes including intellectual disability (ID), epilepsy, strabismus, and other neurological or somatic features [42,43,56,57].

In our case, the identified *NEXMIF* c.1882C>T (p.Arg628*) pathogenic variant is classified as pathogenic and introduces a premature stop codon, likely leading to the loss of function. Interestingly, all previously reported cases with this specific pathogenic variant have been females. Our patient exhibited a complex neurodevelopmental phenotype, including ASD, ADHD, mild ID, and epilepsy—features that align with previously described *NEXMIF*-associated disorders [42,58].

### 4.2. NEXMIF and the Sex-Dependent Expression of X-Linked Disorders

One of the most intriguing aspects of *NEXMIF*-related disorders is the sex-based variability in phenotype severity. Males typically exhibit more severe clinical manifestations due to the hemizygous state of the X chromosome, while females show a broader phenotypic spectrum, likely influenced by X-inactivation, mosaicism, and cellular interference [59].

Several studies have demonstrated that while males with *NEXMIF* variants frequently present with profound ID and autistic traits, females can exhibit a range of phenotypes from mild cognitive impairment to significant neurodevelopmental deficits, often with epilepsy [45,52]. Our case further supports this heterogeneity as our female patient exhibited significant neurodevelopmental impairment despite previous reports suggesting a generally milder phenotype in females.

Webster et al. (2017) suggested that skewed X-inactivation may account for why some female carriers manifest severe symptoms, while others remain asymptomatic. Additionally, recent data indicate that the type of *NEXMIF* pathogenic variant (nonsense, missense, frameshift, or deletions) may influence clinical severity [56]. The c.1882C>T (p.Arg628*) variant identified in our patient appears to correlate with a more severe neurodevelopmental presentation, which could be attributed to a lack of residual protein function.

### 4.3. NEXMIF and Epilepsy: Expanding the Clinical Spectrum

In addition to ASD and ID, epilepsy is a frequent feature in *NEXMIF*-related disorders. De Lange et al. (2016) reported that 12 out of 14 females with *NEXMIF* variants exhibited different types of epilepsy, highlighting the strong association between *NEXMIF* dysfunction and seizure susceptibility. The types of seizures described include myoclonic, absence, and atonic seizures, reinforcing the role of NEXMIF in cortical excitability and network stability [42,51].

A notable aspect of our case is the presence of Jeavons syndrome, a generalized epilepsy characterized by eyelid myoclonia, absence seizures, and photosensitivity. The genetic contribution to Jeavons syndrome continues to be actively studied. In a 2021 review, Mayo et al. identified several candidate genes potentially associated with eyelid myoclonia with absence, including *SYNGAP1*, *KIAA2022/NEXMIF*, *RORB*, and *CHD2*, while pathogenic variants in *SLC2A1*, *KCNB1*, and *NAA10* were also suggested to have possible implications [31]. Given its involvement in generalized epileptic encephalopathies, *NEXMIF* dysfunction may contribute to the broader spectrum of these disorders, including Jeavons syndrome. However, further studies are required to determine whether Jeavons syndrome represents a recurrent phenotype in *NEXMIF*-related epilepsy.

Interestingly, Wang et al. (2023) described a *NEXMIF* pathogenic variant (c.937C>T; p.R313) in a patient with multi-organ failure and epilepsy, suggesting that *NEXMIF* dysfunction might have systemic implications beyond the central nervous system [53]. While our patient did not exhibit multi-organ involvement, she was overweight, consistent with Langley et al. (2022), who proposed that obesity might be an additional feature of *NEXMIF*-related disorders [52].

### 4.4. Clinical Implications and the Need for Genetic Screening

Our findings reinforce the growing recognition that *NEXMIF* variants contribute to a wide phenotypic spectrum, necessitating a high index of suspicion for genetic testing in patients presenting with neurodevelopmental disorders, particularly those with ASD and epilepsy. The genetic heterogeneity of ASD makes the early identification of contributory variants crucial for guiding prognosis, genetic counseling, and, potentially, personalized therapeutic approaches [20,21,60].

Given the variability in *NEXMIF*-related disorders, standardized genetic screening guidelines should be considered for patients with overlapping ASD, ID, and epilepsy phenotypes. The early detection of *NEXMIF* variants could aid in risk stratification and help clinicians anticipate potential complications, such as seizure severity or cognitive impairment.

Skewed X-inactivation analysis could be considered for our patient. This test is usually used for the analysis and diagnosis of X-linked conditions. The process of X-inactivation is of great importance for the expression of a genetic disease. Both symptomatic and asymptomatic females may have X-chromosome inactivation skewed patterns. Additionally, if the patient’s relatives decide to do the genetic testing, it can determine the appropriate test for them [61,62].

Further research is needed to elucidate the precise molecular mechanisms by which NEXMIF dysfunction contributes to neurodevelopmental disorders. Future studies should explore the following:

- The role of *NEXMIF* in synaptic plasticity and neuronal network formation.
- The relationship between specific *NEXMIF* variants and epilepsy subtypes.
- The impact of X-inactivation patterns on clinical severity in female carriers.

This study expands the current knowledge on *NEXMIF* variants by providing one of the first documented cases of Jeavons syndrome associated with a pathogenic *NEXMIF* variant. Our findings highlight the importance of recognizing epilepsy as a major feature of *NEXMIF*-related disorders and suggest that the c.1882C>T (p.Arg628*) pathogenic variant may be associated with a more severe phenotype in females than previously assumed.

Given the growing evidence of *NEXMIF*-associated phenotypic variability, clinicians should consider comprehensive genetic testing in patients with ASD and epilepsy, particularly those with early-onset seizures and cognitive impairment. As genetic screening becomes more widely available, further studies will be essential to refine genotype–phenotype correlations and optimize management strategies for *NEXMIF*-related disorders [63].

## 5. Conclusions

This study reinforces the critical role of genetic testing in the early and accurate diagnosis of complex neurodevelopmental disorders. The identification of a rare *NEXMIF* pathogenic variant (c.1882C>T; p.Arg628*) in our patient, combined with a detailed review of the literature, expands the current understanding of *NEXMIF*-related encephalopathy and its associated phenotypic variability.

Our case supports the hypothesis that *NEXMIF* variants contribute significantly to the pathogenesis of autism spectrum disorder (ASD), intellectual disability, and epilepsy. The presence of Jeavons syndrome in our patient further highlights the expanding clinical spectrum of *NEXMIF*-associated disorders and underscores the necessity of recognizing epileptic phenotypes in these patients.

Importantly, all previously reported cases with this specific pathogenic variant have been in females, raising questions about the role of X-inactivation, genetic compensation, or mosaicism in modulating phenotypic expression. Given the observed variability in clinical presentation, genetic testing could be considered in patients with unexplained neurodevelopmental disorders, particularly in those with overlapping ASD and epilepsy phenotypes.

The findings from this study highlight the importance of genetic testing for an earlier diagnosis, improved therapeutic strategies, and a better-informed genetic counseling for affected families. Future research should focus on elucidating the precise molecular mechanisms underlying *NEXMIF*-related disorders and exploring potential targeted therapeutic interventions.

## Figures and Tables

**Table 1:** NEXMIF variant p.Arg628* in the GnomAD v4.1.0 database.

Variant ID	Source	HGVS Consequence	Allele Count	Allele Number	Allele Frequency
X-74742674-C-CA	Exome	p.Arg628Leu	1	1210952	8.26 × 10^−7^
X-74742674-C-T	Exome and genome	p.Arg628 Gln	15	1209439	1.24 × 10^−5^
X-74742675-G-T	Exome	p.Arg628Arg	1	1211109	8.26 × 10^−7^

**Table 2:** Cases of c.1882C>T (p.Arg628*) variant.

Reference	Gender, Age (Years)	Pathogenic Variant	Variant of Uncertain Significance	Benign Variant	Clinical Symptoms	Phenotype	Family History
De Lange et al., 2016 [42]	Female 2.3	c.1882C>T p.Arg628*	N/A	N/A	Motor delay, hypotonia, joint laxity speech delay, Mild Intellectual Disability, age of first notice delay—7, no additional medical problems, normal MRI brain	No dysmorphic features described	N/A
Athanasakis E. et al., 2013 [43]	Female 13	c.1882C>T, p.Arg628*	N/A	N/A	XCI—65:35, Mild Intellectual Disability, no data regarding autistic behavior or other neurodevelopmental problems	N/A	Negative for ID

**Table 3:** Overview of *NEXMIF* variants reported between 2017 and 2024: from genetic variants to clinical manifestations.

Reference	Sex	Age (Years)	Method	Genetic Testing	Symptoms
Kozhanova T.V. et al., 2017 [44]	F	5	targeted sequencing	*KIAA2022* gene, exon 3 p.Asp451fs	epilepsy, psychomotor, speech and intellectual development delay, behavioral disorders and autistic symptoms.
Lambert N. et al., 2018 [45]	-	-		*NEXMIF* de novo variant c.3470C>A p.Ser1157*	
Lorenzo M. et al., 2018 [46]	M	19		*KIAA2022* variant c.652C > T p.Arg218*	long ears, anteverted nares, keratoconus, strabismus, a narrow forehead, thick vermilion of the upper and lower lips, macroglossia, prognathism, café-au-lait spots, gastroesophageal reflux, gastrointestinal problems, difficulty regulating temperature, musculoskeletal impairments, one grand mal seizure.
	M	10		novel nonsense de novo variant c.2707G > T p.Glu903*	mild dysmorphic features, axial hypotonia, gastrointestinal problems, visual impairment, strabismus, difficulty regulating temperature, cutaneous mastocytosis.
Meyers C. et al., 2018 [47]	-	-		*KIAA2022* p.R322*	epilepsy with myoclonic–atonic seizures.
Kholin A.A., Kholina E.A., 2020 [48]	M	21	DNA sequencing	de novo microdeletion of four nucleotides in *KIAA2022* gene: c.1713_1716del p.Ser571ArgfsTer13	muscular dystonia/hypotonia, motor delay, polymorphic seizures, modified MRI: predominant frontal cortical atrophy, ventriculomegaly.
Cioclu M.C. et al., 2021 [49]	F	28	NGS exome sequencing on genomic DNA	*NEXMIF* c.2171delG p.S724MfsTer5	absences with eyelid myoclonia seizures, modified MRI: thinning middle frontal gyrus of the prefrontal cortex, temporal lobe cortex, pericalcarine visual cortex.
Ogasawara M. et al., 2020 [50]	F	46	trio whole-exome	heterozygous de novo pathogenic variant, *NEXMIF* c.1123del p.Glu375Argfs*21	prognathism, thick lower lip, open mouth, depressed nasal bridge, speech delay, Mild Intellectual Disability, generalized tonic–clonic seizures, obesity.
Chorny L.E. et al., 2022 [51]	M	8		*NEXMIF* c.2478_2479dup	generalized myoclonic epilepsy, progressive cognitive decline.
Langley E. et al., 2022 [52]	M	13	proband exome sequencing	*NEXMIF* c.788delC p.T2631fsX41	motor delay, nonverbal, autism, intellectual disability, constipation, proteinuria, allergy, hypothyroidism, tonic–clonic seizures.
	M	20 months	trio, whole exome sequencing + mitochondrial sequencing	*NEXMIF* c.846_849delTGTC p.V283TfsX20	left eye strabismus, hyperpigmentation spots, axial hypotonia, obesity, motor delay, speech delay, intellectual delay, autism symptoms.
	F	6	epilepsy panel	possibly mosaic, de novo pathogenic variant *NEXMIF* Arg333*	square face, long palpebral fissures, a short philtrum, thin upper lip, learning disability, speech delay, agitation, aggression, anxiety, Attention Deficit/Hyperactive Disorder absence seizures.
Wang L. et al., 2023 [53]	F	3	whole exome sequencing	heterozygous de novo variant *NEXMIF* c.937C>T p.R313*	Epileptic seizures, MOF, disseminated intravascular coagulation, hemophagocytic syndrome.
Aygün et al., 2024 [54]	F	5	next generation sequencing technology (NGS)	*NEXMIF* c.45512_4513del p. Phe1505*	facial dysmorphism, cutaneous malformations, ocular malformations, anal atresia, distal hypotonia, scoliosis.
Qi H. et al., 2024 [55]	M	8 months	whole exome sequencing (WES)	*NEXMIF* c.1042C > T p. Arg348	strabismus, motor delay, language delay, intellectual delay, seizures, pulmonary anomalies, obesity.

## Data Availability

All data generated or analyzed are included in the article.
